# Microbiological characteristics of different tongue coatings in adults

**DOI:** 10.1186/s12866-022-02626-7

**Published:** 2022-09-09

**Authors:** Caihong He, Qiaoyun Liao, Peng Fu, Jinyou Li, Xinxiu Zhao, Qin Zhang, Qifeng Gui

**Affiliations:** 1 Department of Geriatrics, The First Affiliated Hospital, School of Medicine, Zhejiang University, Hangzhou, Zhejiang China; 2 Key Laboratory of Diagnosis and Treatment of Aging and Physic-Chemical Injury Diseases of Zhejiang Province, The First Affiliated Hospital, School of Medicine, Zhejiang University, Hangzhou, Zhejiang China; 3 First Affiliated Hospital, Heilongjiang University of Chinese Medicine, Harbin, China

**Keywords:** Tongue coating, Microbiome, Co-occurrence networks, Driver species, Disease prevention

## Abstract

**Background:**

Tongue coating is an important health indicator in traditional Chinese medicine (TCM). The tongue coating microbiome can distinguish disease patients from healthy controls. To study the relationship between different types of tongue coatings and health, we analyzed the species composition of different types of tongue coatings and the co-occurrence relationships between microorganisms in Chinese adults.

From June 2019 to October 2020, 158 adults from Hangzhou and Shaoxing City, Zhejiang Province, were enrolled. We classified the TCM tongue coatings into four different types: thin white tongue fur (TWF), thin yellow tongue fur (TYF), white greasy tongue fur (WGF), and yellow greasy tongue fur (YGF). Tongue coating specimens were collected and used for 16S rRNA gene sequencing using the Illumina MiSeq system. Wilcoxon rank-sum and permutational multivariate analysis of variance tests were used to analyze the data. The microbial networks in the four types of tongue coatings were inferred independently using sparse inverse covariance estimation for ecological association inference.

**Results:**

The microbial composition was similar among the different tongue coatings; however, the abundance of microorganisms differed. TWF had a higher abundance of *Fusobacterium periodonticum* and *Neisseria mucosa*, the highest α-diversity, and a highly connected community (average degree = 3.59, average closeness centrality = 0.33). TYF had the lowest α-diversity, but the most species in the co-occurrence network diagram (number of nodes = 88). The platelet-to-lymphocyte ratio (PLR) was associated with tongue coating (*P* = 0.035), and the YGF and TYF groups had higher PLR values. In the co-occurrence network, *Aggregatibacter segnis* was the “driver species” of the TWF and TYF groups and correlated with C-reactive protein (*P* < 0.05). *Streptococcus anginosus* was the “driver species” in the YGF and TWF groups and was positively correlated with body mass index and weight (*P* < 0.05).

**Conclusion:**

Different tongue coatings have similar microbial compositions but different abundances of certain bacteria. The co-occurrence of microorganisms in the different tongue coatings also varies. The significance of different tongue coatings in TCM theory is consistent with the characteristics and roles of the corresponding tongue-coating microbes. This further supports considering tongue coating as a risk factor for disease.

## Background

Microbial-host interactions affect the health status of humans [1]. The microbiome defends us against pathogens, contributes to the development of the immune system, and helps metabolize various compounds [2, 3]. The microbiota is reportedly altered in various conditions such as obesity, schizophrenia, autism, and Parkinson's disease [4]. Tongue coating is an important indicator used in traditional Chinese medicine (TCM) to diagnose diseases. In TCM theory, it is believed that tongue coating can reflect the condition of the body's *zang* and *fu* (internal organs), *qi-xue*, and fluids as well as the nature and severity of disease [5]. Previous studies have suggested a link between the TCM tongue coating classification and disease. Wang et al. [6] systematically reviewed the tongue coatings of patients with coronavirus disease of 2019 (COVID-19) and found a higher incidence of greasy coatings than other coatings. Lo et al. [7] found that the tongue coating thickness of early-stage breast cancer patients is different from that of healthy people. A study on the improvement of sleep quality with Cheonwangbosim-dan (CWBSD) found that improved sleep was associated with increased tongue moss at the root of the tongue, and the abundance of *Veillonella* was altered [8].

There are different types of tongue coatings in healthy people [9]. Therefore, it is important to clarify the relationship between tongue coating types and tongue microecology in healthy people for disease prevention using TCM. Chen et al. [10] classified the tongue coatings of 94 healthy people into eight categories according to the theory of TCM and found that there was no significant difference in the overall composition of tongue-coating microbiota among different types of tongue coatings. However, they only analyzed the differences in microbial composition and abundance between different tongue coatings, without comparing the co-occurrence of microorganisms or establishing associations between microorganisms and clinical indicators.

In this study, we classified tongue coatings into four types according to color, thickness, and moistness: thin white tongue fur (TWF), thin yellow tongue fur (TYF), white greasy tongue fur (WGF), and yellow greasy tongue fur (YGF). This classification was used to evaluate the microbiota composition by 16sRNA sequencing. We not only analyzed the compositional differences of microorganisms in different tongue coatings, but also the co-occurrence relationship between microorganisms. Additionally, we evaluated the biological indicators of the population in different tongue coatings and established a link between microorganisms and clinical indicators. This will help healthcare professionals estimate overall health using changes in tongue coatings enabling them to choose appropriate treatments in the early disease stages to prevent disease progression.

## Results

### Participant characterization

**Fig. 1:**
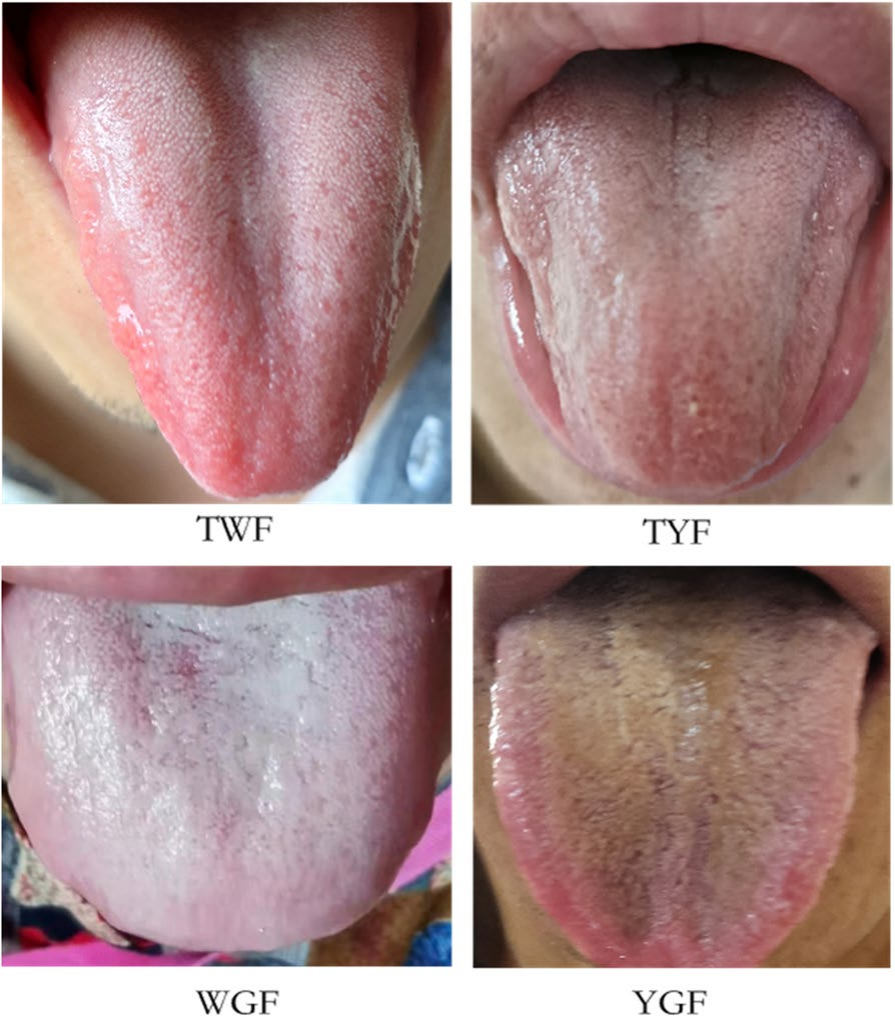
Chinese medicine tongue coating types and photos. Tongue coating: a layer of moss-like material covering the tongue. TWF:thin white tongue fur; TYF:thin yellow tongue fur; WGF:white greasy tongue fur; YGF:yellow greasy tongue fur

Examples of the four tongue coatings are shown in Fig. 1. The participants’ clinical information is summarized in Table 1. A total of 158 participants were included in this study, the average age was 48 years, including 78 men and 80 women. According to the TCM tongue examination, the number of participants unique to YGF, WGF, TWF, and TYF were 41,48,55 and 14. There was no statistically significant difference between groups in each clinical indicator, except for the platelet-to-lymphocyte ratio (PLR). The PLR is an inflammatory indicator, and its value was the highest in the TYF group, followed by the YGF group. The difference in PLR values among the four groups was statistically significant (*P* = 0.035). This suggested that inflammation was most pronounced in the TYF and YGF groups.

**Table 1:** Clinical characteristics for all participants

Variables	TWF (*n *= 55)	TYF (*n* = 14)	WGF (*n* = 48)	YGF (*n* = 41)	*p*
Age, Median (IQR)	41.0 (32.0, 58.0)	54.0 (36.5, 57.0)	52.0 (33.0,63.0)	49.0(42.8,60.5)	0.31
Sex, n (%)					0.149
Men	27 (49.1)	3 (21.4)	27 (56.2)	21 (51.2)	
Women	28 (50.9)	11 (78.6)	21 (43.8)	20 (48.8)	
Education, n (%)					0.271
Primary school and below	10	7	11	13	
Middle school	9	3	15	13	
Graduate and above	30	3	17	14	
Smoking, n (%)					0.405
Yes	14 (29.2)	1 (9.1)	15 (35.7)	11 (28.2)	
No	34 (70.8)	10 (90.9)	27 (64.3)	28 (71.8)	
Drinking, n (%)					0.413
Yes	10 (20.8)	1 (9.1)	6 (14.6)	11 (28.2)	
No	38 (79.2)	10 (90.9)	35 (85.4)	28 (71.8)	
Hypertension, n (%)					0.304
Yes	4 (9.5)	2 (15.4)	10 (25)	6 (15.8)	
No	38 (90.5)	11 (84.6)	30 (75)	32 (84.2)	
Diabetes, n (%)					0.823
Yes	1 (2.2)	0 (0)	0 (0)	1 (2.6)	
No	45 (97.8)	13 (100)	40 (100)	38 (97.4)	
Place, n (%)					0.215
City	31 (70.5)	5 (38.5)	24 (60)	24 (60)	
Country	13 (29.5)	8 (61.5)	16 (40)	16 (40)	
NLR, Median (IQR)	1.8 (1.5, 2.4)	1.6 (1.5, 2.1)	1.6 (1.3, 2.2)	1.8 (1.5, 2.2)	0.494
CRP, Median (IQR)	0.5 (0.3, 1.2)	2.7 (2.0, 4.7)	0.5 (0.2, 1.5)	0.7 (0.3, 1.4)	0.071
PLR, Mean ± SD	119.6 ± 28.9	142.9 ± 35.5	110.4 ± 35.8	123.2 ± 29.4	0.035*

*NLR* Neutrophil to lymphocyte ratio, *CRP* C-reactive protein, *PLR* Platelet-to-lymphocyte ratio, n Number. Data in bold indicate statistically significant values; **p < 0.001, *p < 0.05

### Tongue coating microbial a-diversity and β-diversity analysis among the four groups

**Fig. 2:**
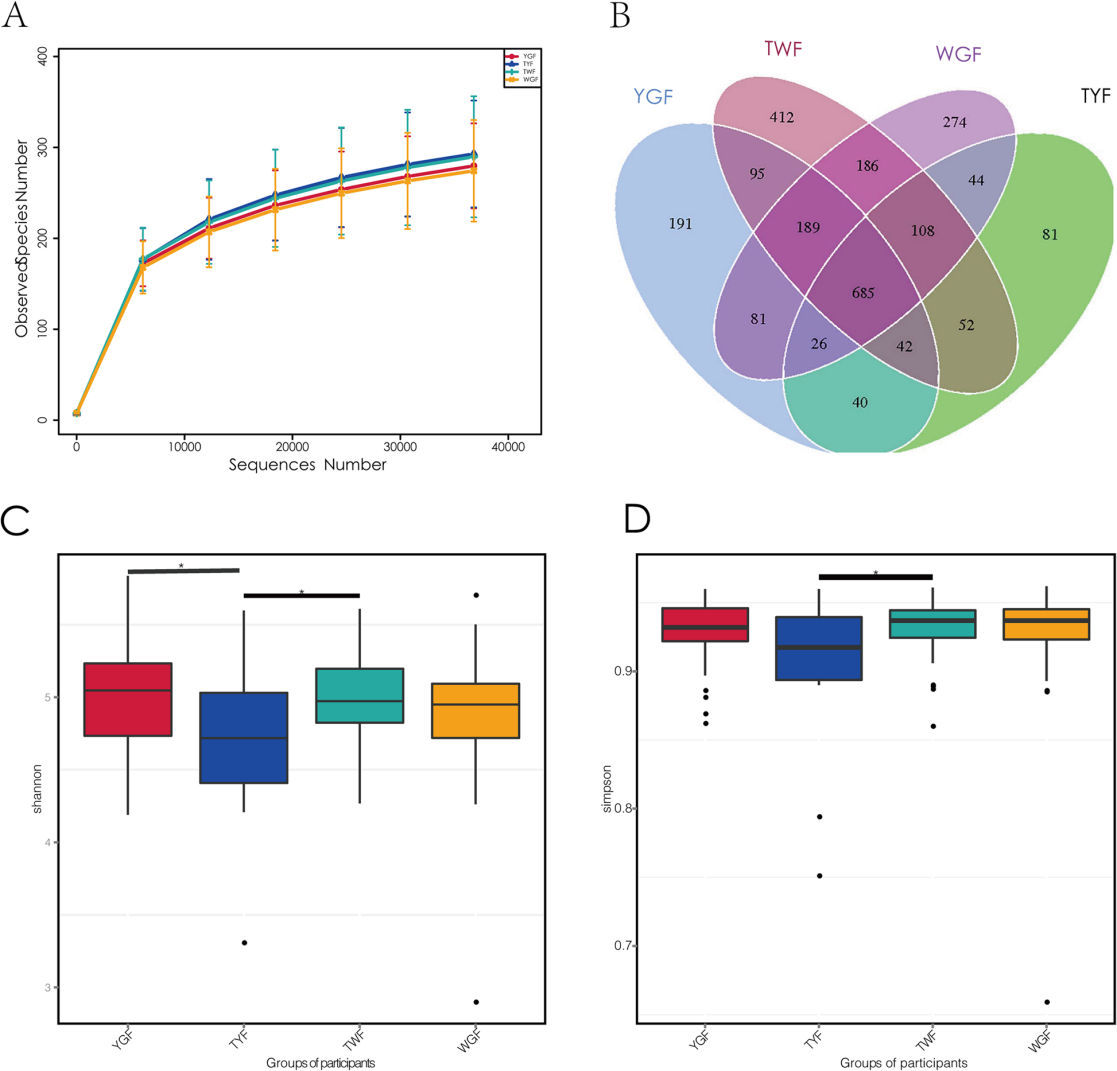
Analysis of microbial a-diversity of four different types of tongue coating. **A** Rarefaction curve: the abscissa is the number of sequencing strips randomly selected from a sample, and the ordinate is the number of OTUs that can be constructed based on the number of sequencing strips, which is used to reflect the sequencing depth. Different samples are represented by curves of different colors. **B** Venn diagram of OTUs with four different tongue coatings.The Veen diagram depicts the number of OTUs specific and shared by each of the four tongue coatings TWF, TYF, YGF, and WGF. **C** and **D** Results of a-diversity analysis of four tongue coatings. **C** Simpson index; **D** Shannon index

Based on the Illumina Nova sequencing platform data, an average of 98,448 tags were sequenced per sample, and an average of 94,175 valid data points were obtained after quality control, with 62,441 valid data points in quality control and 63.71% quality control efficiency. The sequences were clustered into operational taxonomic units (OTUs) with 97% identity, and 2,518 OTUs were obtained, among which 2.314 (91.90%) could be annotated to the SILVA 138 database for species,at the boundary (91.90%), phylum (72.44%), family (67.67%), genus (57.59%), and species levels (20.97%). According to the rarefaction data (Fig. 2A), the rarefaction curve had plateaued, indicating that almost all microorganisms of the four tongue coatings were detected at the selected sequencing depth.

Venn diagram analysis revealed that 685 OTUs were common to all groups, and the number of OTUs unique to YGF, WGF, TWF, and TYF were 191, 274, 412, and 81, respectively. This indicating differences in OTUs among the four groups (Fig. 2B). To investigate the difference in a-diversity among groups, we calculated six a-diversity indices, including Chao1, ACE, Shannon, Simpson, Goods coverage, and observed species richness. The results showed that the TWF group had higher Shannon (*P* = 0.037) and Simpson (*P* = 0.046) indices than the TYF group (Fig. 2C), and that the YGF group had a higher Shannon index than the TYF group (*P* = 0.045) (Fig. 2D). As shown in Fig. 2C, TYF had the lowest a-diversity.

**Fig. 3:**
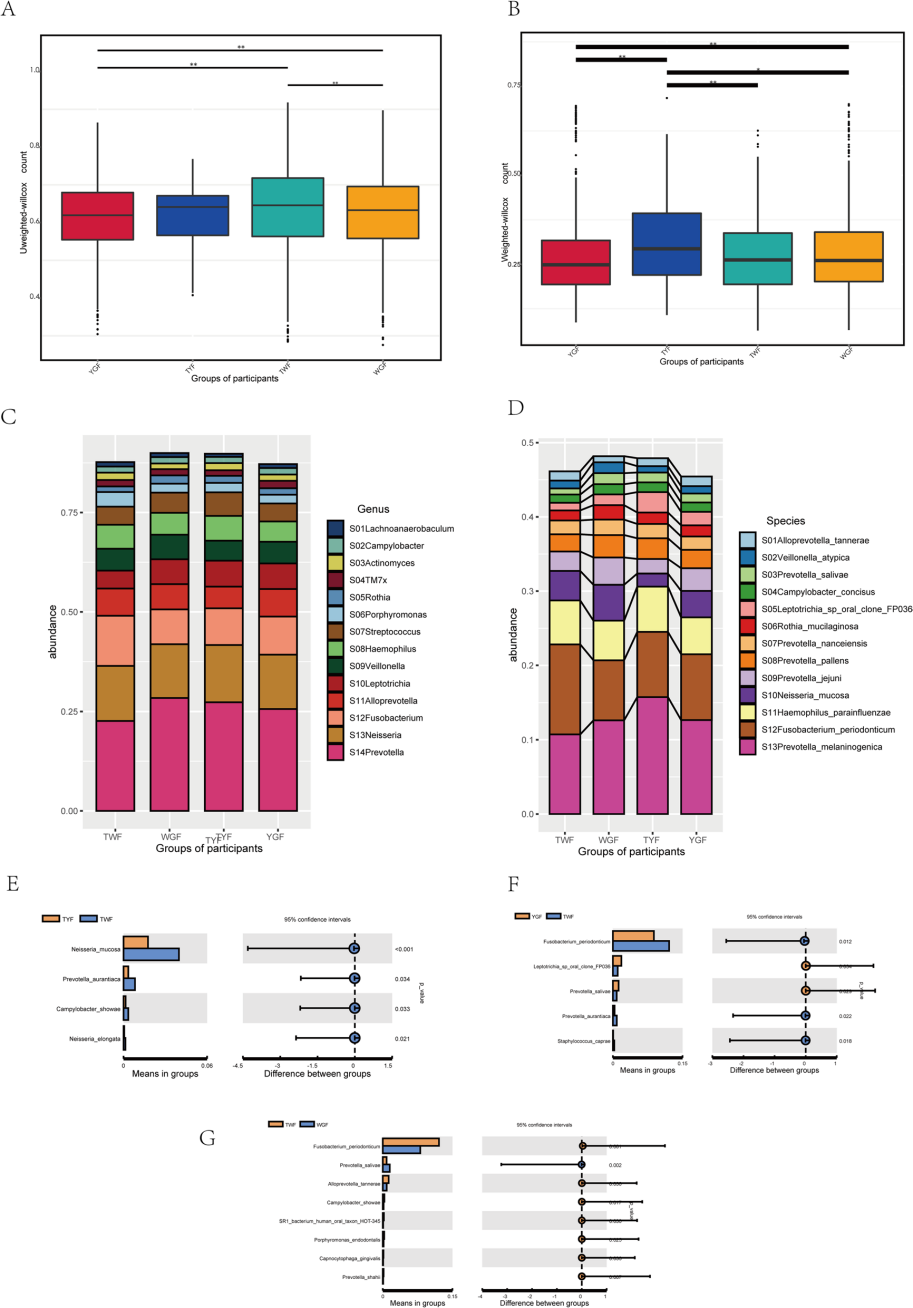
Analysis of microbial β-diversity of four different types of tongue coatings **A **and** B** Results of β-diversity analysis of four tongue coatings. **A** unweighted Unifrac distances and (**B**) weighted Unifrac distances. **C **and** D** Cumulative graph of microorganisms with percentages greater than 1 in four different tongue coatings. **C** Genus level and (**D**) Species level. **E **and** F** The differences in microbial abundance between TWF and the remaining three tongue coating types at the species level showed statistically significant results. **E** TWF-TYF, (**F**) TWF-YGF and (**G**) TWF-WGF

For the β-diversity analysis, we found statistically significant differences between the YGF and TYF groups (*p* < 0.001) and between the TWF and TYF groups (*P* = 0.003) using weighted UniFrac distances (Fig. 3 A and B). We next compared microbial taxonomic differences among groups. We found similar microbial community components among the four tongue coatings and investigated the differential taxonomic abundance among groups (Fig. 3 C and D). TCM theory considers thin white coating as a normal tongue manifestation; therefore, we compared the microbial abundance of tongue coating in the TYF, WGF, and YGF groups with that in the TWF group at the species level (Fig. 3 E–G). We found that the TWF group had a higher abundance of *Fusobacterium periodonticum* (*P* < 0.05) and a lower abundance of *Prevotella salivae* (*P* < 0.05) than the WGF and YGF groups. The abundance of *Neisseria mucosa* was significantly higher in the TWF group than in the TYF group (*P* < 0.001).

### Symbiosis of tongue coating microorganisms among four groups of tongue coatings

Although the abundance of certain microbes may contribute to a specific phenotype in a given environment, the interrelationship between microorganisms also plays an important role in the phenotype [11, 12]. We thus performed a co-occurrence network analysis to predict microbial interactions among the four groups using sparse inverse covariance estimation for ecological association inference (SPIEC-EASI). Although TYF had the lowest a-diversity, it did not affect the number of species involved in the co-occurrence network. As shown in Table 2, the TYF group had the most species involved in the co-occurrence network diagram (number of nodes: 88). The WGF group had the most mutually promoting relationships and the least mutually inhibiting relationships between the species (positive edges: 103; negative edges: 13). The TWF network had the highest number of connections per node (average degree: 3.59) and the lowest average closeness centrality (0.33), indicating a highly connected community (Table 2). In contrast, the TYF network had the lowest number of connections per node (average degree: 2.02) and a high average closeness centrality (0.54), indicating a poorly connected community (Table 2).

**Table 2:** Correlations and topological properties of the four tongue coatings microbiome networks

Network properties	TWF	TYF	YGF	WGF
Number of nodes	64	88	84	80
Number of edges	115	89	116	116
Positive edges	90	66	96	103
Negative edges	25	23	20	13
Modularity	5.28	14.70	15.25	10.36
Average degree	3.59	2.02	2.76	2.90
Average closness centrality	0.33	0.54	0.57	0.35

Number of connections/corelations obtained by SPIEC-EASI analysis

**Fig. 4:**
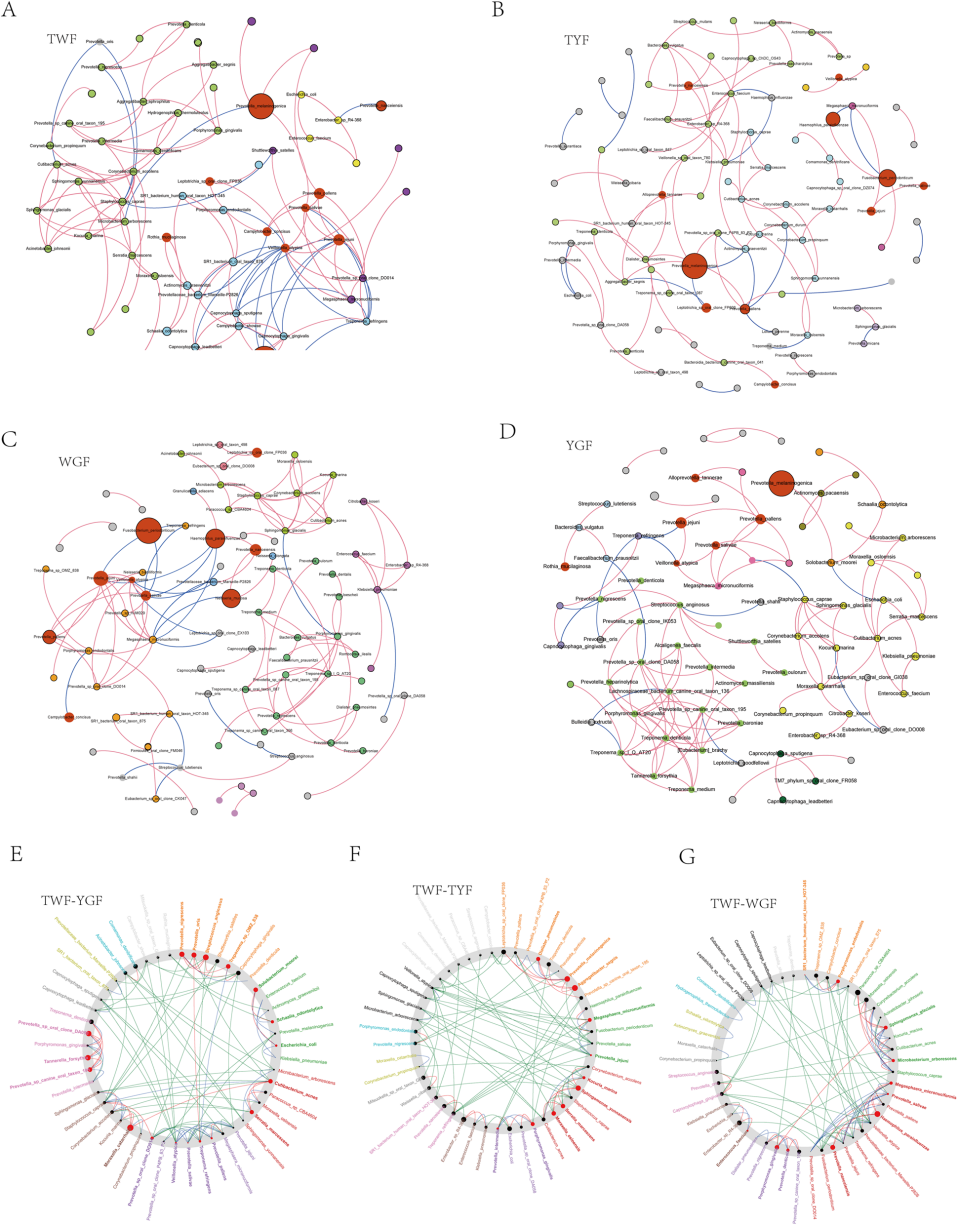
Discrepancies of co-occurrence networks of the four different tongue coating types **A**, **B**, **C** and **D** The complex nature of inter microbial interactions in the ecological community of each tongue coating was characterized by co-occurrence networks using graphs. Main groups of co-occurrence species are presented in different colours, and smaller groups are shown in grey. Networks of species in the four tongue coatings are identified by positive and negative correlations among the dominant bacteria. Red line indicates positive correlation and blue line indicates negative correlation. Only the bacterial connections (edges) larger than cut-offs (correlation values > 0.4) are retained. Each node in the network indicates a species. The size of each node is proportional to the relative abundance of each species. Nodes in red color show driver microbes which significantly contributed to the separation of the networks (NESH-score value of > 2). **E, F** and **G** Network view: All nodes belonging to the same community are randomly assigned similar colors, and gray nodes represent nodes existing in both case and control subnets. The node size is directly proportional to its nesh score. If the nesh score of a node is case > control, it will be marked in red. Therefore, a large red node can be regarded as a driving node. For edges, red indicates the interaction that exists only in the case subnet, green indicates the interaction that exists only in the control subnet, and blue indicates the interaction that exists in both subnets

We used ‘NetShift’ to predict driver microbes that facilitate community changes in microbial association networks among groups (Fig. 4). Interestingly, further evaluation of the driver microbes between TYF and TWF revealed *Aggregatibacter segnis*, *Prevotella melaninogenica*, *Sphingomonas yunnanensis* and *Enterobacter spp. R4-368* as the top four critical nodes, with a high NESH score (NESH-score > 2). The network with the TWF group as a control and WGF as a case showed that *Haemophilus parainfluenzae* and *Paracoccus spp.* and *CBA4604* were the driver microbes with high NESH scores (NESH-score > 2). Evaluating the driver microbes between YGF and TWF showed that *Moraxella catarrhalis*, *Streptococcus anginosus*, *Prevotella spp. oral clone DA058*, and *Tannerella forsythia* had a high NESH score (NESH-score > 2).

### Association of key taxa with clinical indicators

**Fig. 5:**
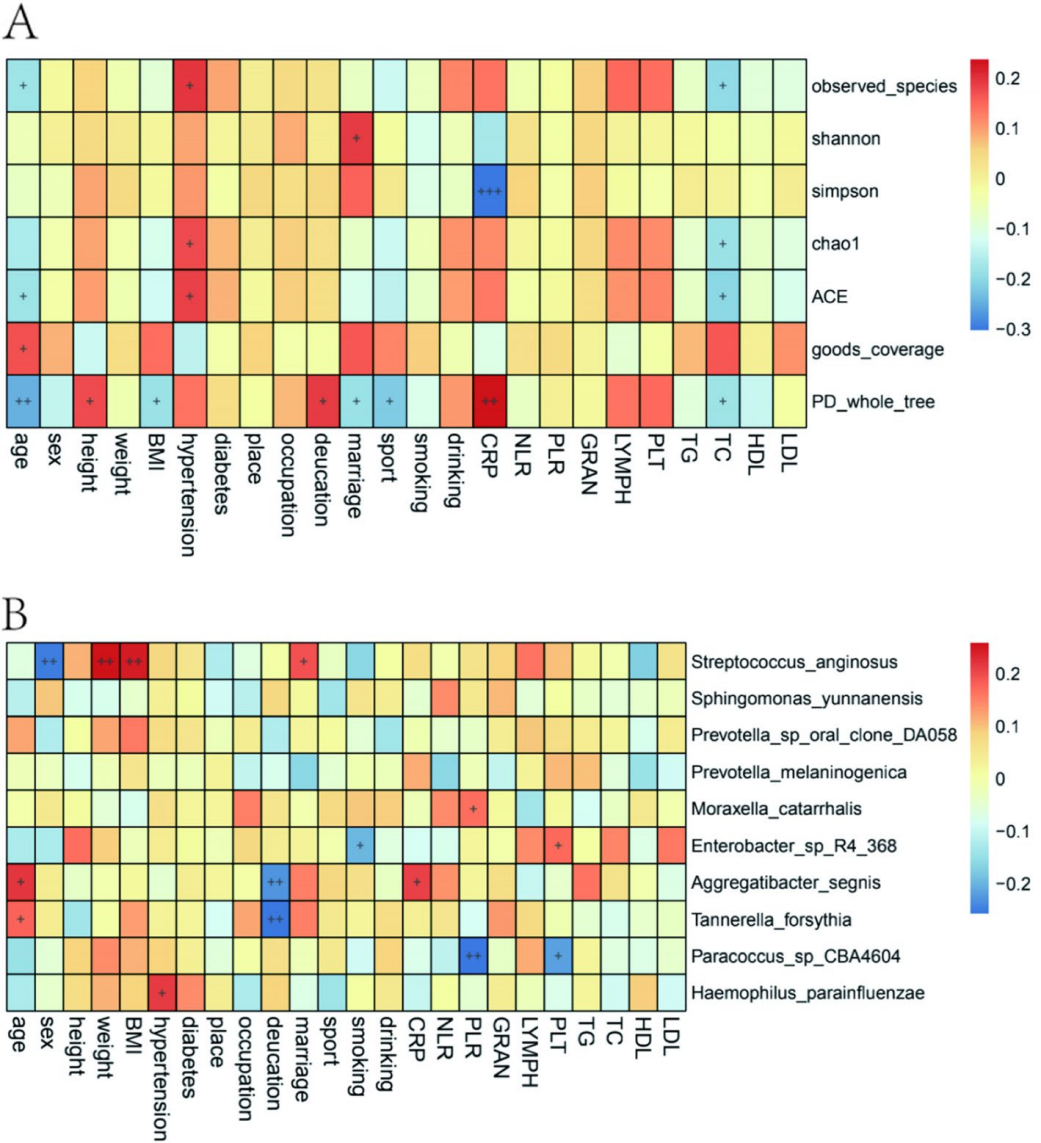
**A** The effect of clinical indicators and demographic variables on the microbial a-diversity of the tongue, with red representing positive correlations and blue representing negative correlations.” + ” indicates *p* < 0.05,” +  + ” indicates *p* < 0.001. **B** Contributions of clinical and demographic variables to the differences in relative abundances of key distinguishing bacterial species for four tongue coatings based on Spearman correlation coefficients and best multiple regression model. Color bar shows correlation values, where red color indicates positive association, blue color negative association, and only significant correlations were shown.” + ” indicates *p* < 0.05,” +  + ” indicates *p* < 0.001

To further analyze the associations between clinical indicators and altered tongue microbiota, we performed a correlation analysis between a-diversity indices and C-reactive protein (CRP), serum total cholesterol, body mass index (BMI), age, and blood pressure, which had a significant effect on the complexity of the flora. CRP was negatively correlated with simple diversity (*P* < 0.001) and positively correlated with whole-tree phylogenetic diversity (PD) (*P* < 0.05), indicating that as CRP increased, the diversity of the flora decreased, but the PD increased. Age was negatively correlated with observed_species, ACE, and whole-tree PD, and positively correlated with goods coverage (Fig. 5A).

We next analyzed the correlation between clinical indicators and the driver microbes identified by NetShift analysis, including *Streptococcus anginosus*, *Sphingomonas yunnanensis*, *Prevotella sp. oral clone DA058*, *Prevotella melaninogenica*, *Moraxella catarrhalis*, *Enterobacter sp*. *R4-368*, *Aggregatibacter segnis*, *Tannerella forsythia*, *Paracoccus sp. CBA4604* and *Haemophilus spp* (Fig. 5B). We found that the relative abundance of *Aggregatibacter segnis* was correlated with CRP and age (*P* < 0.05), and negatively correlated with education (*P* < 0.001) and that *Aggregatibacter segnis was* the driver microbe between TYF and TWF. The abundance of *Streptococcus anginosus* was positively correlated with BMI and weight and was the driver microbe between YGF and TWF. PLR as an indicator of inflammation positively correlated with the abundance of *Moraxella catarrhalis* and negatively with the abundance of *Paracoccus sp CBA4604*. *Moraxella catarrhalis* was the driver microbe between YGF and TWF, and *Paracoccus sp CBA4604* was the driver microbe between WGF and TWF.

## Discussion

In TCM theory, different tongue coatings represent different symptoms, although not always associated with a disease state, but as a risk factor for certain diseases, similar to constitutional medicine. In some studies of the correlation between tongue coating and *Helicobacter pylori* (Hp) infection and gastroscopic manifestation, Hp infection was found to be most common in yellow greasy coating [13]. Cyanotic tongue, yellow coating and spotted tongue were associated with both gastric antrum mucosal hyperemia and edema [14]. These results suggest that yellow greasy coating and other phenomena may be risk factors for gastritis, which should be recognized and prevented in advance. Our study revealed differences in the abundance of some species in different tongue coatings showed co-occurrence between species, and indicated the correlation of microbes with clinical indicators. This provides a basis for subsequent studies on the use of tongue coating as a risk factor for disease prediction.

a -diversity analysis indicated that the TYF group had the lowest Shannon and Simpson indices, and that other a -diversity indices were not significantly different between tongue coatings. β-diversity analysis showed that the TYF group differed from the YGF and TWF groups and that the differences between the other groups were not statistically significant. This may be related to the fact that our study subjects were relatively healthy and the number of participants in the TYF group was relatively small.

Comparison of microorganisms between different tongue coatings should not be limited to a comparison of microbial composition and abundance, but should also focus on the co-occurrence network. A global survey of gut microecological composition and interaction networks in different regions and age groups found that North Americans and Europeans have similar gut microecological compositions but different patterns in their microbial interaction networks [15]. Microbes in a community can be mutually beneficial and interdependent, and can also help each other evade the host immune system [16]. Conversely, microorganisms compete with each other for a common source of nutrients [17, 18]. In many processes, key microbial groups are likely to act as ‘drivers’ [19, 20], facilitating several changes and becoming an important factor for understanding the microbial basis of a disease. Interestingly, the driver microbes in the interaction networks are not necessarily the most abundant taxa, but play an important role in maintaining the connectivity and function of the network [21]. TWF is considered a healthy coating on the tongue [22], and TWF from healthy people has been used as a control in many studies [23–25]. This study found that TWF networks exhibited the highest number of connections per node (mean degree: 3.59) and lowest mean closeness centrality (0.33), indicating a highly connected community. In addition, the TWF network presented a modular structure characterized by the presence of different groups of nodes with high numbers of interconnections within the groups, and a certain degree of independence between groups [26]. A highly connected and modular microbiome can be more efficient in resource consumption and can reduce the success rate of pathogen invasion [27, 28]. TWF had a higher abundance of *Fusobacterium periodonticum* and *Neisseria mucosa* than the other three tongue coatings. *Fusobacterium periodonticum* is a normal organism in the oral cavity, and its abundance increases rapidly in the first 2 years of life [29]. The nasopharyngeal microbiome characteristics of COVID-19 patients showed a significant decrease in the abundance of *Fusobacterium periodonticum* [30]. Yoneda et al. [31] and Mormiroli et al. [32] reported that *Fusobacterium periodonticum* is involved in the surface sialylation process. The sialome (the broad variety of sialic acid compounds in the human body) also plays a defensive role against viral infections since a reduction in sialic metabolism can increase susceptibility to severe acute respiratory syndrome coronavirus 2. *Neisseria mucosa* is a normal colonizing bacterium commonly found in the oral cavity and nasopharynx; however, in rare conditions it can also cause infectious diseases, such as meningitis and endocarditis [33, 34].

We generated a microbial association network for the TYF (’case’) and TWF (’control’) sets and applied the NetShift workflow. *Aggregatibacter segnis*, *Prevotella melaninogenica*, *Sphingomonas yunnanensis*, and *Enterobacter sp R4-368* were considered the driver microbes between the ‘case’ and ‘control’. Figure 5B shows that *Aggregatibacter segnis* was positively correlated with CRP and age and negatively correlated with education. *Aggregatibacter segnis* is one of the most abundant bacteria in oral squamous cell carcinoma tissues [35] and exhibit increased abundance in patients with bronchial asthma [36]. *Prevotella melaninogenica* is a gram-negative anaerobic commensal bacterium that resides in the oral cavity and upper respiratory tract and is associated with periodontal disease and aspiration pneumonia [36]. In TCM theory, TYF indicates the initial stage of heat, mostly seen in the syndrome of wind-heat invading the lung [37] (similar to upper respiratory tract infections in modern medicine). Previous studies found that the neutrophil count in the yellow coating group was significantly higher than that in the white coating group [38]; therefore, we speculate that *Aggregatibacter segnis* and *Prevotella melaninogenica* may be responsible for the switch from TWF to TYF. Our results are inconsistent with those of Ye et al. [25], who suggested that *Bacillus* is closely associated with typical yellow tongue coating and is a potential diagnostic marker for it. This may be related to inconsistencies in our study population. *Streptococcus anginosus* was considered one of the ‘driver microbes’ between the YGF (‘case’) and TWF (‘control’), and was positively correlated with BMI and weight. *Streptococcus anginosus* is a gram-positive bacterium that is present in the mouth, upper respiratory tract, gastrointestinal tract, and vagina as a normal inhabitant. It can cause severe invasive infections [39] and is a risk factor for esophageal cancer [40]. TCM theory considers YGF as a common tongue coating for patients with phlegm dampness and heat, and phlegm dampness is associated with obesity [41]. This tongue coating is common in patients with nutritional, metabolic, and gastrointestinal diseases such as diabetic nephropathy and chronic gastritis [42–44]. Therefore, we speculate that *Streptococcus anginosus* can be used as a monitoring indicator for improving YGF-related diseases in TCM, and a follow-up study should further elucidate this mechanism. In this study, we also found higher PLR values in patients in the TYF and YGF groups. PLR is an indicator often used to respond to inflammation [45]. This result is consistent with the observation that the ‘driver microbes’ of TYF and YGF were associated with inflammation. *Haemophilus parainfluenzae* was a ‘driver microbe’ between the WGF (‘case’) and the TWF (‘control’) groups. This bacterium is a gram-negative bacterium and is a normal inhabitant of the human respiratory tract. However it is associated with a variety of infectious diseases under certain conditions, such as infective endocarditis, pneumonia, otitis media and arthritis [46, 47]. WGF is frequently observed in patients with acute cerebral infarction and is associated with fibrinogen levels [38].

According to TCM theory, highly skilled physicians can detect and treat disease risk factors before the disease state is established to cure the disease at an early stage or prevent its development. The presence of specific microbial features in each tongue coating may predict potential dysfunction of specific organs and the development of specific diseases. Therefore, different types of tongue coatings may serve as risk factors, requiring further research using large sample sizes. There are some limitations to this study. For example, the population in this study was relatively healthy; the traditional clinical indexes do not reflect the health differences between different tongue coating populations and do not reflect that the TWF group populations are the healthiest. Therefore, in future research, we will perform metabolomics and immunomarker testing of tongue coatings as well as blood analysis to further clarify the health differences between different tongue coating populations and their relationship with disease.

## Conclusion

In TCM theory, tongue coating is an important basis for diagnosing diseases, representing health status, predicting disease prognosis, and playing an important role in the diagnosis and treatment of patients with COVID-19. Tongue coating microbiota is a highly stable and important component of the oral microbiota. Tongue coating microorganisms, are involved in a variety of metabolic mechanisms, and disorders of tongue coating microorganisms are associated with a variety of diseases. In the present study, the significance of different tongue coatings in TCM theory was consistent with the characteristics and roles of the corresponding tongue-coating microbes. Subsequently, we expect to establish further links between tongue coating and diseases using multi-omics studies or tongue-gut microbial clustering analysis in different disease populations. This research will support the use of tongue coating to predict or diagnose diseases.

## Methods

### Study participants

Healthy adult subjects were recruited from June 2019 to October 2020 from the communities of Hangzhou and Shaoxing City, Zhejiang Province, China. Inclusion criteria for healthy adult subjects were: (i) 18–59 years of age; (ii) good physical condition, with no uncomfortable symptoms or unusual signs; (iii) preservation of daily dietary habits one week before sampling, with no significant changes in diet; (iv) voluntary and signed informed consent; and (v) complete demographic data. The exclusion criteria were: (i) acute infectious diseases (active tuberculosis, respiratory or oral infections, etc.), presence of infection symptoms such as cough and runny nose; (ii) cardiovascular diseases (coronary heart disease, etc.), respiratory diseases (chronic obstructive pulmonary disease, pneumoconiosis, etc.), renal insufficiency, malignancy, digestive system diseases (active hepatitis, inflammatory bowel disease, irritable bowel syndrome, chronic diarrhea, etc.), history of gastrointestinal surgery (gastrointestinal resection, esophageal resection, etc.), neurological diseases (acute cerebrovascular accident, Parkinson's disease, etc.); (iii) use of probiotics, prebiotics, synbiotics, or antibiotics within 8 weeks prior to sampling, and intestinal preparation within 4 weeks; (iv) poorly controlled hypertension, diabetes mellitus, and hyperlipidemia; (v) autoimmune diseases (rheumatoid, oral dryness, etc.); (vi) depression, bipolar disorder, or other psychiatric disorders; (vii) dental or orthodontic surgery within the last 8 weeks, with oral diseases such as oral ulcers and periodontitis; (viii) hepatitis B, hepatitis C, human immunodeficiency virus, or other infections.

Participants completed a general information form, which included height, weight, sex, age, smoking and drinking habits, residence, exercise, and diet. The questionnaire data quality was controlled by investigators professionally trained on the objectives, filling, and administration methods.

### Tongue coating types

A registered Chinese medicine practitioner was on hand to pre-classify the tongue coatings into four types: TWF,TYF,WGF,and,YGF according to the principles of TCM tongue diagnosis. Meanwhile, images of the subjects' tongue coatings were taken under natural light using a smartphone (Huawei P30, Huawei Technologies Co., Ltd., China). The steps for image acquisition included the following [48] (i) the subject was asked to expose the tongue as far as possible so that the complete tongue surface could be photographed; (ii) the AI and filter functions of the smartphone were turned off and the flash function was turned on; (iii) on the front side of the subject, the phone was placed approximately 15 cm away from the tongue and at an angle of 45°to the lips; (iv) the screen was tapped to adjust the focus and then the picture was taken. The two registered Chinese medicine practitioners classified the tongue coatings according to the tongue coating images, selected images with the same results as the on-site Chinese medicine practitioners and retained the tongue-coating specimens.

### Sample collection

Participants were instructed to fast and refrain from brushing their teeth and tongue before sampling. The researchers gently wiped two sterile swabs on the surface of each participants' tongues three times, broke the tips of the swabs, placed them into a 2 mL lyophilized tube, and immediately tightened the nut. The above steps were repeated twice, and the two samples were retained. The samples were frozen in liquid nitrogen and transferred to a − 80 °C refrigerator within 1 h.

### Metagenomic DNA extraction, polymerase chain reaction (PCR) amplification, purification, and library construction

Total metagenomic DNA was extracted from the samples using the cetyltrimethylammonium bromide/ sodium dodecyl sulfate method. DNA concentration and purity were monitored using 1% (w/v) agarose gels. DNA was diluted to 1 ng/µL using sterile water. The PCR products were detected by electrophoresis using a 2% (w/v) agarose gel, and the appropriate PCR products were purified using magnetic beads, quantified by enzyme labeling, mixed in equal amounts, and then detected by electrophoresis using a 2% (w/v) agarose gel. The target bands were recovered using a gel recovery kit provided by Qiagen (Hilden, Germany).

Sequencing libraries were generated using the TruSeq® DNA PCR-Free Sample Preparation Kit (Illumina, San Diego, CA, USA) following the manufacturer's recommendations and adding index codes. V3-4 hypervariable regions of the bacterial 16S region. Library quality was assessed on a Qubit 2.0 Fluorometer (Thermo Fisher Scientific, Waltham, MA, USA) and an Agilent Bioanalyzer 2100 system. Finally, the library was sequenced on an Illumina NovaSeq platform and 250 bp paired-end reads were generated.

### Bioinformatics analysis

Raw tags were obtained by splicing the reads with overlap using the Flash software (V1.2.7, http://ccb.jhu.edu/software/FLASH/) [49]. Quality filtering of the raw tags was performed under specific filtering conditions to obtain high-quality clean tags according to the QIIME (V1.9.1) quality-controlled process [50]. The clean tags were compared with a database (16S:Gold database ITS:Unite database http://drive5.com/uchime/uchime_download.html) to obtain the final effective tag data. The final effective tags were clustered using the Uparse software (Uparse v7.0.1001 http://drive5.com/uparse/), and the sequences were clustered into OTUs with 97% consistency by default. Species annotation was performed on OTU sequences to obtain taxonomic information and to determine the community composition of each sample at each taxonomic level separately.

### Bacterial diversity and co-occurrence networks analysis

Venn diagram construction was performed using the R software (version 2.15.3). The QIIME software (version 1.9.1) was used to calculate Chao1, abundance-based coverage estimator (ACE), Shannon, Simpson, Goods coverage, and observed species indices. The R software was used to analyze the differences in a-diversity between groups,and Unifrac distances were calculated using the QIIME software; microbial networks in the tongue coatings were inferred independently using SPIEC-EASI and implemented using the Spiec-Easi R package [51]. We used ‘NetShift’ to predict driver microbes that facilitate community changes in microbial association networks among groups.

## Data Availability

The datasets generated and analysed during the current study are available in the [Genome Sequence Archive (GSA)] repository,[https://ngdc.cncb.ac.cn/gsa/s/91m0cDLk].

## Abbreviations

BMI: Body mass index
COVID-19: Coronavirus disease 2019
CRP: C-reactive protein
PCR: Polymerase chain reaction
PD: Phylogenetic diversity
PERMANOVA: Permutational multivariate analysis of variance
PLR: Platelet-to-lymphocyte ratio
SARS-CoV-2: Severe acute respiratory syndrome coronavirus 2
SPIEC-EASI: Sparse inverse covariance estimation for ecological association inference
TC: Total cholesterol
TCM: Traditional Chinese medicine
TWF: Thin white tongue fur
TYF: Thin yellow tongue fur
WGF: White greasy tongue fur
YGF: Yellow greasy tongue fur
